# Supplementary material for: Patterns of Intron Gain and Loss in Fungi
Source: PLoS Biol. 2004 Nov 30;2(12):e422. doi: 10.1371/journal.pbio.0020422 (PMC532390; doi:10.1371/journal.pbio.0020422)
Supplement: Table S1 — Also available at http://genes.mit.edu/NielsenEtAl/. (4.3 MB ZIP). [file pbio.0020422.st001.zip › NielsenEtAl/html/525.html]

AN6717.1.NCU04899.1.MG09367.1.FG02461.1


```
 CLUSTAL W (1.82) Multiple Sequence Alignments - Introns Inserted


Sequence 1: MG09367.1	336 aa
Sequence 2: FG02461.1	336 aa
Sequence 3: NCU04899.1	336 aa
Sequence 4: AN6717.1	358 aa
Alignment Length: 360 aa
Number Identitical Residues: 237 aa
Alignment Score (without introns) 10004


MG09367.1 	MFAASR----IQTRAFSAAARN0LSKVTVLGAAGGIGQPLSLLLKLNPRVTELALYDIRG
NCU04899.1	MFAASR----IQTRAFSASARQ0LTKVSVLGAAGGIGQPLSLLLKLNPRVSELALYDIRG
FG02461.1 	MFAASR----IQRRAFSATARD0LSKVTVLGAAGGIGQPLSLLLKMNPRVTDLALYDIRG
AN6717.1  	MFAARQSFGFLQKRAFSASASQ0ASKVAVLGASGGIGQPLSLLLKLNPRVSELALYDIRG
          	**** :: . :* *****:* :  :**:****:************:****::********

MG09367.1 	GPG~VAADISHINTKSNVKGYDPTPSGLAAALKGSEVVLIPAGVPRKPGMTRD-------
NCU04899.1	APG~VGADLSHINTKSTVKGYEPTASGLADALKGSEIVLIPAGVPRKPGMTRD-------
FG02461.1 	GPG1VAADISHVNTKSSVKGYEPNAAGLKEALSGAEVVLIPAGVPRKPGMTRD-------
AN6717.1  	GPG1VAADISHINTNSTVKGYEPTESGLADALKGSEIVLIPAGVPRKPGMTRDGKEDDCE
          	.** *.**:**:**:*.****:*. :**  **.*:*:****************.......

MG09367.1 	-----------DLFNTNASIVRDLAKACAESCPEANILVISNP~VNSTVPICAEVFKARG
NCU04899.1	-----------DLFNTNASIVRDLAKACAESCPEANILVISNP~VNSTVPIVSEIFKKAG
FG02461.1 	-----------DLFNTNASIVRDLAKAAAEAAPKAKLLIISNP~VNSTVPIVKEVYKAAG
AN6717.1  	NLRIGTDLLMTDLFNTNASIVRDLAKAAAKASPEANILVISNP0VNSTVPIVSEVFKAAG
          	.   .:.   :****************.*::.*:*::*:**** *******  *::*  *

MG09367.1 	VYNPKRLFGVTTLDVVRASRFVSEIKGSDPKDENITVVGGHSGVTIVPLFSQSNHPDLS-
NCU04899.1	VYNPKRLFGVTTLDVVRASRFVSEIKGTDPKDENITVVGGHSGVTIVPLFSQSKHPELS-
FG02461.1 	VYNPKTLFGVTTLDVVRASRFVSEIKGTDPKDENITVVGGHSGVTIVPLFSQSNHPDLS-
AN6717.1  	VYNPKRLFGVTTLDVVRASRFISQVQGTDPSKEAVPVVGGHSGVTIVPLLSQSNHPNIDG
          	***** ***************:*:::*:**..* :.*************:***:**::..

MG09367.1 	-ANDQLVNRVQFGGDEVVKAKDGAGSATLSMAMAGARMAESVLRAAQGEKGVIEPTFVDS
NCU04899.1	-KNEQLIHRVQFGGDEVVKAKDGAGSATLSMAMAGARMAESLLRAAQGEKGVIEPTFVDS
FG02461.1 	-SNAELVKRVQFGGDEVVKAKDGAGSATLSMAMAGARMADSVLRAVQGEKGVKEPAFVES
AN6717.1  	KTRDELVHRIQFGGDEVVKAKDGAGSATLSMAMAGARFAESLLRAAQGEKGVVEPTFVES
          	. . :*::*:***************************:*:*:***.****** **:**:*

MG09367.1 	PLYKDQGIEFFSSKVELGPNGVEKILPIGEIDANEQKLLDACVGDLKKNIEKGVAFVASN
NCU04899.1	PLYKDQGIDFFASKVELGPNGVEKIYPVGPVDEVEQKLLDACLVDLKKNIQKGKDFVAAN
FG02461.1 	PLYKDQGIEFFSSQVELGPEGVEKIHPLGKLDANEEKLVDAALVDLKKNIEKGVAFVASN
AN6717.1  	PLYKDQGVNFFASKVELGPNGAEKINPVGEVNEFEQKLLEACLVDLKKNIQKGIDFVKAN
          	*******::**:*:*****:*.*** *:* ::  *:**::*.: ******:**  ** :*

MG09367.1 	PGK
NCU04899.1	PGK
FG02461.1 	PPK
AN6717.1  	P--
          	*
```
